# Supplementary material for: Trends in the Prescription of Non-Vitamin K Antagonist Oral Anticoagulants for Atrial Fibrillation: Results of the Polish Atrial Fibrillation (POL-AF) Registry
Source: J Clin Med. 2020 Nov 5;9(11):3565. doi: 10.3390/jcm9113565 (PMC7694480; doi:10.3390/jcm9113565)
Supplement: Supplementary file 1 [file jcm-09-03565-s001.pdf]

Supplementary Table S1. Prescription pattern of apixaban, dabigatran and rivaroxaban across centres.

number of centre	type of centre	all n=2971	Apixaban n=953	Dabigatran n=819	Rivaroxaban n=1199
1	academic	709	267 (37.7)	221 (31.2)	221 (31.2)
2	academic	284	101 (35.6)	68 (23.9)	115 (40.5)
3	academic	151	51 (33.8)	37 (24.5)	63 (41.7)
4	regional	272	119 (43.8)	82 (30.1)	71 (26.1)
5	academic	351	65 (18.5)	97 (27.6)	189 (53.8)
6	academic	60	15 (25.0)	7 (11.7)	38 (63.3)
7	military	430	132 (30.7)	73 (17.0)	225 (52.3)
8	regional	248	72 (29.0)	76 (30.6)	100 (40.4)
9	academic	257	93 (36.2)	91 (35.4)	73 (28.4)
10	academic	209	38 (18.2)	67 (32.0)	104 (49.8)

Supplementary Table S2. Factors associated with the selection of individual NOAC over another NOAC for stroke prevention in patients with AF - univariable logistic regression models.

Factors	Apixaban			Dabigatran			Rivaroxaban		
	**OR**	**95%CI**	**p**	**OR**	**95%CI**	**p**	**OR**	**95%CI**	**p**
CHA_2DS_2-VASc score	1.14	1.09-1.19	**<0.0001**	0.94	0.9-0.99	**0.0114**	0.93	0.9-0.97	**0.0008**
HAS-BLED score	1.42	1.3-1.55	**<0.0001**	0.93	0.85-1.02	0.1114	0.77	0.71-0.84	**<0.0001**
Age (per 5 years)	1.19	1.14-1.23	**<0.0001**	0.92	0.89-0.95	**<0.0001**	0.93	0.9-0.96	**<0.0001**
Heart failure	1.37	1.16-1.61	**0.0002**	0.82	0.69-0.97	**0.0187**	0.89	0.77-1.04	0.1493
Vascular disease	1.21	1.03-1.41	**0.018**	1.03	0.88-1.22	0.6852	0.82	0.71-0.95	**0.0088**
Female	1.28	1.1-1.5	**0.0018**	0.73	0.62-0.86	**0.0001**	1.04	0.9-1.2	0.6158
Non-permanent AF	0.65	0.54-0.77	**<0.0001**	1.13	0.93-1.37	0.2112	1.37	1.15-1.63	**0.0005**
Malignancy	1.48	1.04-2.11	**0.0282**	0.77	0.51-1.17	0.2216	0.84	0.58-1.2	0.3256
Any previous bleeding	2.59	1.86-3.62	**<0.0001**	0.81	0.55-1.2	0.2965	0.41	0.28-0.61	**<0.0001**
GFR < 60 ml/min/1.73m^2	1.95	1.66-2.28	**<0.0001**	0.69	0.59-0.81	**<0.0001**	0.75	0.65-0.87	**0.0001**
Antiplatelets with NOAC	2.37	1.58-3.55	**<0.0001**	0.62	0.38-1.04	0.0678	0.58	0.37-0.91	**0.0168**

Abbreviation: CI, confidence interval; eGFR, estimated glomerular filtration rate; NOAC, non-vitamin K antagonist oral anticoagulant; OR, odds ratio.
